# Antegrade metallic stent placement using a slim cholangioscope for malignant afferent loop obstruction

**DOI:** 10.1055/a-2387-4238

**Published:** 2024-09-04

**Authors:** Haruo Miwa, Ritsuko Oishi, Kazuki Endo, Hiromi Tsuchiya, Akihiro Funaoka, Yuichi Suzuki, Shin Maeda

**Affiliations:** 1 26437 Gastroenterological Center, Yokohama City University Medical Center, Yokohama, Japan; 2 Department of Gastroenterology, Yokohama City University, Graduate School of Medicine, Yokohama, Japan

Enteral metallic stent placement using balloon enteroscopy is reported as a therapeutic strategy for malignant afferent loop obstruction 1 2 ; however, it is challenging when the enteroscope cannot reach the stricture. Metallic stent placement via endoscopic ultrasound-guided hepaticogastrostomy (EUS-HGS) is rarely reported in such difficult conditions 3 4 . Here we report antegrade metallic stenting using a slim cholangioscope for malignant afferent loop obstruction ( Video 1 ).

Antegrade metallic stent placement for malignant afferent loop obstruction was performed under guidance of a slim cholangioscope.Video 1

A 78-year-old man who underwent pancreaticoduodenectomy with Roux-en-Y reconstruction was referred to our hospital with a benign hepaticojejunostomy anastomotic stricture. EUS-HGS was performed because balloon enteroscopy could not reach the afferent loop ( Fig. 1 ). The patient developed cholangitis 2 years later due to a malignant afferent loop obstruction with abdominal dissemination ( Fig. 2 ). Enteral metallic stent placement was performed via the EUS-HGS route ( Fig. 3 ). An endoscopic retrograde cholangiopancreatography catheter was inserted into the afferent loop; however, a guidewire could not pass through the stricture. A slim cholangioscope (9-Fr eyeMAX; Micro-Tech, Nanjing, China) was inserted into the afferent loop via the EUS-HGS and hepaticojejunostomy anastomoses. The guidewire was then successfully passed through the stricture using a cholangioscope. Contrast-enhanced imaging revealed a localized stricture in the afferent loop. After cholangioscope removal, a balloon dilation catheter was inserted; however, it could not pass through the stricture. A guide sheath (UMIDAS sheath cannula; UMIDAS Inc., Kanagawa, Japan) was inserted into the stricture and a biliary metallic stent with an ultraslim delivery system (YABUSAME Neo; KANEKA Medics, Tokyo, Japan) was successfully passed and placed through the guide sheath. Contrast agent flowed out through the metallic stent and the patient’s abdominal pain was relieved.

**Fig. 1:**
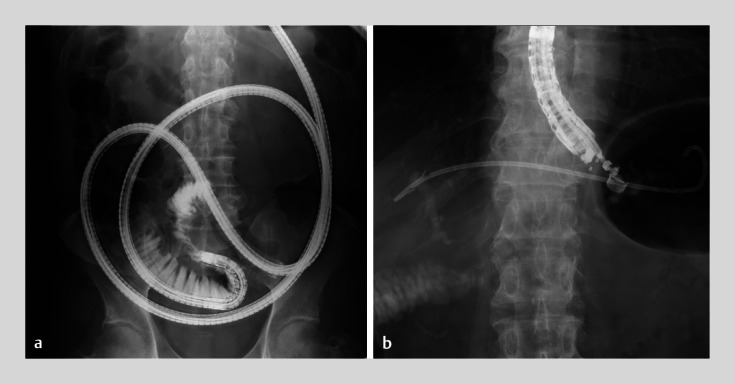
Fluoroscopic images. **a** A single-balloon enteroscope could not reach into the afferent loop. **b** Endoscopic ultrasound-guided hepaticogastrostomy was performed for hepaticojejunostomy anastomotic stricture.

**Fig. 2:**
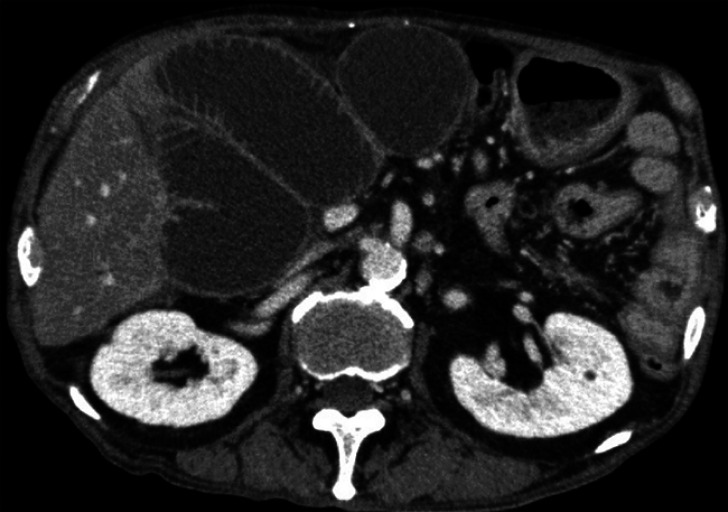
Computed tomography showed a dilated afferent loop due to a malignant stricture.

**Fig. 3:**
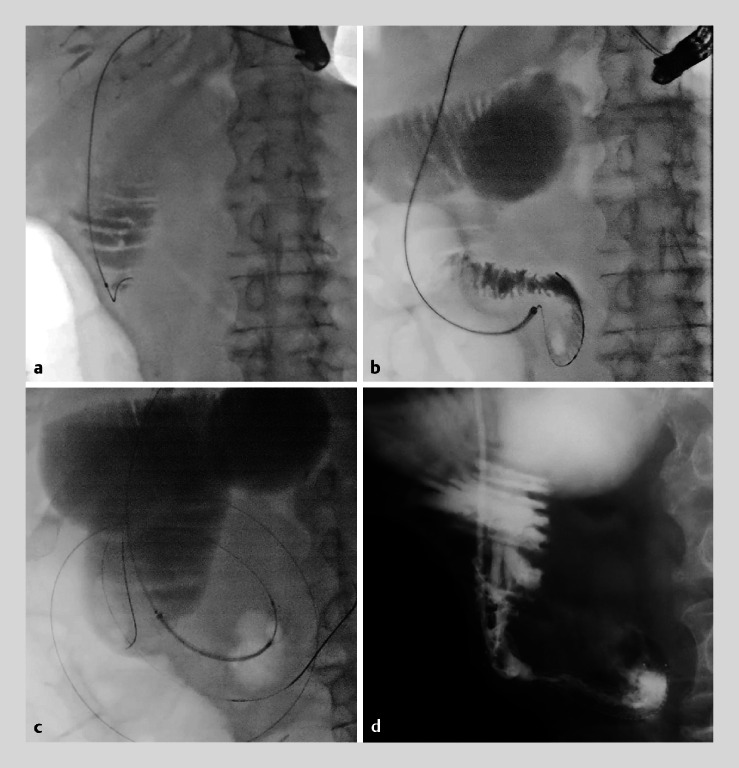
Fluoroscopic images. **a** An endoscopic retrograde cholangiopancreatography catheter was inserted into the afferent loop and the stricture was revealed. **b** The guidewire was passed through the stricture under guidance of the slim cholangioscope. **c** A metallic stent with ultraslim delivery was inserted through the guide sheath. **d** The stent was successfully placed, and the contrast agent flowed out.

To the best of our knowledge, this is the first report of antegrade metallic stent placement using cholangioscopy in a case of malignant afferent loop obstruction. This case demonstrates that a slim cholangioscope may aid guidewire placement in difficult conditions.

Endoscopy_UCTN_Code_TTT_1AR_2AZ

## Notes

Endoscopy E-Videos https://eref.thieme.de/e-videos
*E-Videos* is an open access online section of the journal *Endoscopy* , reporting on interesting cases and new techniques in gastroenterological endoscopy. All papers include a high-quality video and are published with a Creative Commons CC-BY license. Endoscopy E-Videos qualify for HINARI discounts and waivers and eligibility is automatically checked during the submission process. We grant 100% waivers to articles whose corresponding authors are based in Group A countries and 50% waivers to those who are based in Group B countries as classified by Research4Life (see: https://www.research4life.org/access/eligibility/ ). This section has its own submission website at https://mc.manuscriptcentral.com/e-videos .
